# Risk factors for *Cryptosporidium* infection in low and middle income countries: A systematic review and meta-analysis

**DOI:** 10.1371/journal.pntd.0006553

**Published:** 2018-06-07

**Authors:** Maha Bouzid, Erica Kintz, Paul R. Hunter

**Affiliations:** 1 Norwich Medical School, University of East Anglia, Norwich, United Kingdom; 2 NIHR Health Protection Research Unit in Gastrointestinal Infections, University of East Anglia, Norwich, United Kingdom; Christian Medical College, Vellore, INDIA

## Abstract

**Background:**

*Cryptosporidium* infection causes gastrointestinal disease and has a worldwide distribution. The highest burden is in developing countries.

**Objectives:**

We sought to conduct a systematic review and meta-analysis to identify *Cryptosporidium* risk factors in Low and Middle Income countries (LMICs).

**Methods:**

Medline Ovid and Scopus databases were searched with no restriction on year or language of publication. All references were screened independently in duplicate and were included if they presented data on at least 3 risk factors. Meta-analyses using random effects models were used to calculate overall estimates for each exposure.

**Results:**

The most frequently reported risk factors in the 15 included studies were overcrowding, household diarrhoea, poor quality drinking water, animal contact, open defecation/ lack of toilet and breastfeeding. The combined odds ratio for animal contact was 1.98 (95%CI: 1.11–3.54) based on 11 studies and for diarrhoea in the household 1.98 (95%CI: 1.13–3.49) based on 4 studies. Open defecation was associated with a pooled odds ratio of 1.82 (95%CI: 1.19–2.8) based on 5 studies. Poor drinking water quality was not associated with a significant *Cryptosporidium* risk, odds ratio 1.06 (95%CI: 0.77–1.47). Breastfeeding was protective with pooled odds ratio 0.4 (95%CI: 0.13–1.22), which was not statistically significant.

**Conclusions:**

Based on the included studies, crowded living conditions, animal contact and open defecation are responsible for the majority of *Cryptosporidium* cases in LMICs. Future studies investigating *Cryptosporidium* risk factors should have a good study design and duration, include appropriate number of cases, select suitable controls, investigate multiple relevant risk factors, fully report data and perform multivariate analysis.

## Author summary

*Cryptosporidium* is a parasite that causes diarrhoea and is transmitted through faecal contamination of water and food. Though it occurs in developed nations, it is much more prevalent in developing countries and is associated with high mortality in children under 2 years old. In this review, we looked at published studies on factors that increase the risk of contracting cryptosporidiosis in Low and Middle Income Countries. These factors could be targeted to limit the transmission of the disease. Based on the selected studies, the most important risk factors identified were contacts with animals and presence of infected people in the household. Open defecation was also contributing to the risk of infection by this parasite transmitted through the faecal oral route. Breastfeeding was protective from the infection. Poor drinking water was not responsible for causing the disease.

## Introduction

*Cryptosporidium* is a protozoan parasite with a worldwide distribution infecting humans and animals. In high income countries, *Cryptosporidium* occasionally causes sizable outbreaks due to contaminated water supplies or food sources [1]. In Low and Middle Income Countries (LMICs), cryptosporidiosis is much more prevalent and is associated with a significant burden of gastrointestinal disease. Cryptosporidiosis is highly prevalent in early childhood, with 45% of children infected before the age of 2 [2]. More recent studies showed higher prevalence rates: 77% in slum dwelling Bangladeshi children [3] and 97% in children under 3 years from a birth cohort in Southern India [4]. A study in children under 2 years old estimated 2.9 million and 4.7 million *Cryptosporidium* infections in sub-Saharan Africa and South Asia, respectively [5]. Cryptosporidiosis is associated with watery diarrhoea persisting for over 2 weeks. This chronicity increases the vulnerability of children in LMICs and is the result of interplay of immune naivety, malnutrition and HIV infection [2]. *Cryptosporidium* infection in children in LMICs is associated with malnutrition, stunted growth, and cognitive impairment [6]. Cryptosporidiosis exacerbates malnutrition and is more severe in malnourished subjects [7]. *Cryptosporidium* is the second cause of severe diarrhoea in children under 5 years old in sub-Saharan Africa and south Asia and the leading cause of mortality in children aged 12–23 months [8]. In immunocompromised people such as HIV-positive and transplant patients, cryptosporidiosis is more severe and could result in high mortality rates [1]. The disease burden in both developed and developing countries is likely to be underestimated, due to a large number of asymptomatic or self-limiting diarrhoeal cases, lack of systematic diagnosis of etiologic agent of diarrhoeal disease and reliance on microscopy for routine clinical detection, which is associated with low specificity and sensitivity.

Due to the significant burden of cryptosporidiosis, several studies attempted to elucidate *Cryptosporidium* transmission pathways and risk factors [1, 7, 9–11]. The two *Cryptosporidium* species causing the majority of human infections are *C*. *parvum* and *C*. *homini*s. The former is transmitted mainly through a zoonotic cycle between humans and animals, while the latter is predominately anthroponotic. The main *Cryptosporidium* risk factors were summarised in three reviews [7, 10, 11] and are related to drinking contaminated water, contact with infected animals or humans (particularly children), consumption of contaminated food, recreational use of contaminated water and travel to disease endemic areas. However, these reviews focused on developed countries and one on the USA. To our knowledge, no such review of cryptosporidiosis risk factors in LMICs exists. Therefore, we attempted to address this gap and conducted a systematic review and meta-analysis of higher quality studies investigating risk factors for *Cryptosporidium* infection in LMICs.

## Materials and methods

### Search strategy

The methodology and reporting were in accordance with the “Preferred Reporting Items for Systematic Reviews and Meta-Analyses” (PRISMA) (S1 File). Medline Ovid and Scopus databases were searched with no restriction on year or language of publication up to 12^th^ January 2017. The search strategy was limited to title/ abstract/ keyword using the following MeSH terms/ keywords: (*Cryptosporidium* OR cryptosporidiosis) AND (risk factor OR case control OR cohort OR infection OR sporadic OR prevalence). Reference lists from relevant papers were screened for additional eligible articles.

### Inclusion criteria and data extraction

All references were screened by title and abstract independently in duplicate by MB and EK. Studies investigating *Cryptosporidium* transmission and risk factors were considered for full text analysis and data extraction. Eligibility disagreements were resolved by discussion. Abstracts without full text or complete results section, such as conference proceedings, were excluded. Only studies from LMICs as defined by the Official Development Assistance (ODA) of the Organisation for Economic Co-operation and Development (OECD) were included.

In order to restrict the analysis to higher quality studies, additional inclusion criteria were applied at full text analysis stage: at least 20 *Cryptosporidium* infections were reported and the study assessed at least three relevant risk factors. Examples of irrelevant risk factors: age, gender, rural/urban living, stunting, malnutrition (arguably potential cause and consequence of cryptosporidiosis), household income and type of dwelling, as these could not be directly targeted by preventive public health strategies.

For each article, the following information was extracted: location of the study, duration, type of study, *Cryptosporidium* detection method, age range of participants, number of cases, number of controls (if applicable), selection criteria for cases and controls, risk factors (exposures) investigated and odds ratios (or relative risk or hazard ratio) as reported by the authors or calculated from data presented in the paper (when available).

### Quality assessment

The Newcastle-Ottawa scale (NOS) was used to provide a quality assessment score for all included studies [12]. Case control studies were judged across three domains: selection of cases and controls, comparability of cases and controls and ascertainment of exposure. Cohort studies were judged across three domains: selection of cohorts, comparability of cohorts and assessment of outcome. Cross-sectional studies were assessed as per case control studies. Details of subdomains assessed within each criterion are provided in S2 File. A study is awarded a maximum of one star for each subdomain. For this systematic review, only one star was given for the comparability domain as opposed to two possible stars for the traditional Newcastle Ottawa Scale. Therefore, a maximum of 4 stars for selection, 1 star for comparability and 3 stars for exposure/outcome could be awarded, totalling 8 stars if all factors included in the NOS were unlikely to introduce bias. The studies were considered of high quality if NOS score was 6–8 stars, moderate quality for a score of 3–5 stars and of poor quality if the NOS score was 0–2.

### Data synthesis

The risk factors identified in each study were pooled in a table and categorised. The number of risk factors and the proportion achieving statistical significance were noted. Both univariate and multivariate risk factors were extracted. When at least four papers reported on a particular risk factor, a meta-analysis was performed to calculate a combined random effect odds ratio for this exposure using Reference Manager software (RevMan) [13]. Any available (or calculated) risk factor was included in the meta-analysis regardless of significance. As the majority of studies reported on univariate estimates of risk factors, only these were considered when calculating pooled odds ratios (OR_pooled_). Funnel plots generated using RevMan were used to assess publication bias through visual assessment.

Population attributable fraction (PAF) was calculated using the formula PAF = Pe_pooled_ x [(OR_pooled_-1)/ OR_pooled_)] [14]. Pe_pooled_ = proportion of source population exposed to the risk factor, was calculated if the number of exposed cases and total number of cases was available from at least 50% of the studies used to calculate the pooled odds ratio. Pe_pooled_ calculations were performed in OpenMeta [Analyst] software [15], entering the data as untransformed proportions and performing a binary random effects meta-analysis.

## Results

**Fig 1:**
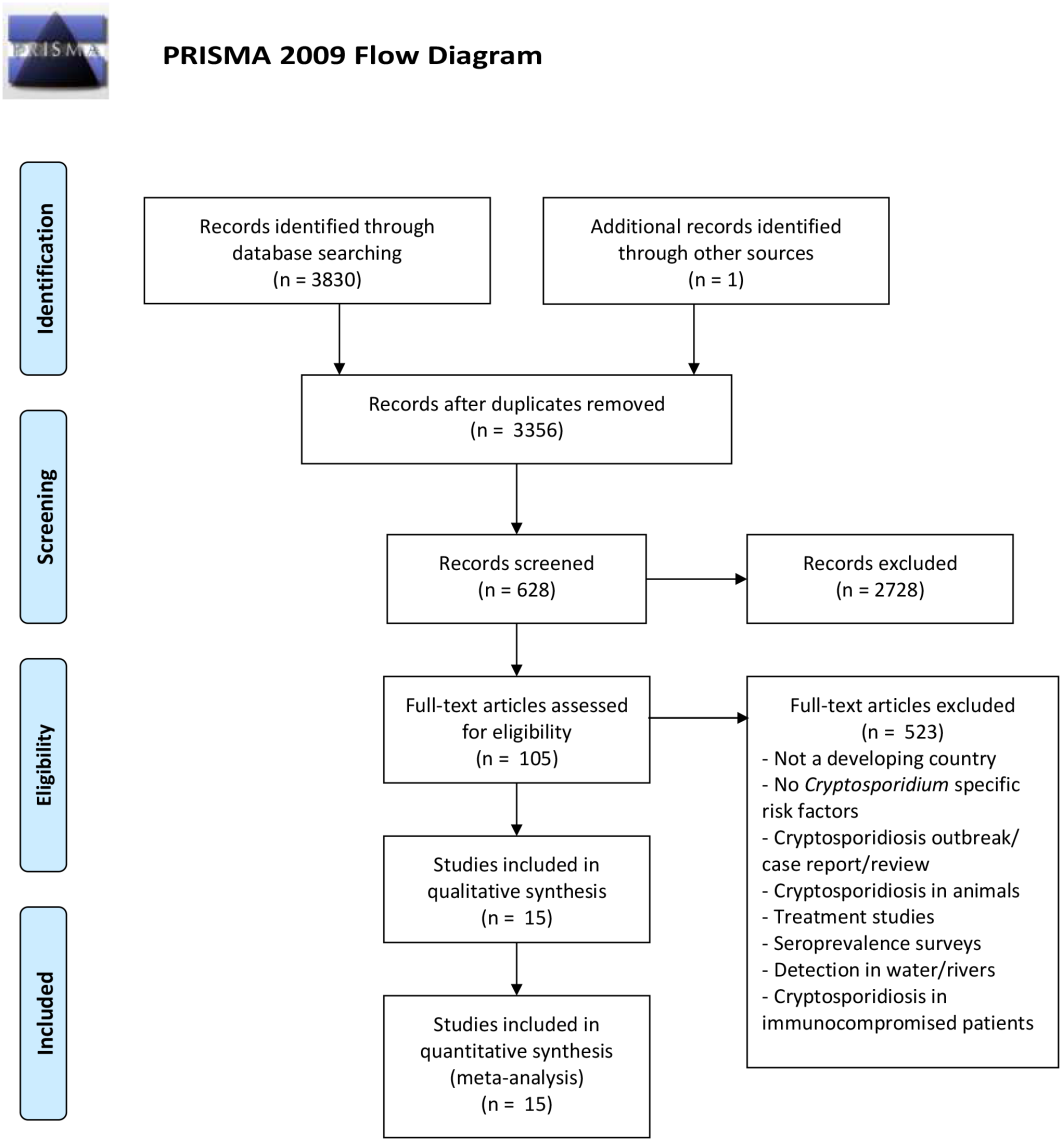
PRISMA flow diagram for peer-reviewed literature search and included studies. *From*: Moher D, Liberati A, Tetzlaff J, Altman DG, The PRISMA Group (2009). *P*referred *R*eporting *I*tems for *S*ystematic Reviews and *Me*ta-*A*nalyses: The PRISMA Statement. PLoS Med 6(7): e1000097. doi: 10.1371/journal.pmed1000097. **For more information, visit** www.prisma-statement.org.

The combined search retrieved 3830 studies, which was reduced to 3356 after duplicate removal (Fig 1). Based on title screening, 627 papers were retained for potential inclusion. An additional study was found by screening reference lists. Therefore, 628 studies were subjected to abstract screening, of which 105 were retained for full text analysis (Fig 1). 523 papers were excluded because of the following reasons: not a developing country, no *Cryptosporidium* specific risk factors, cryptosporidiosis outbreak/ case report, review, cryptosporidiosis in animals, treatment studies, seroprevalence surveys, detection in water/rivers, and cryptosporidiosis in immunocompromised patients.

A total of 15 papers were included for meta-analysis (Fig 1). Table 1 summarises the characteristics of these studies. Six case control studies, 4 cohort studies and 4 cross-sectional studies are included, together with one paper that used case control design for hospital study and cross-sectional design for community study. The quality assessment using NOS showed that 7/15 studies were of high quality, while 6 studies were of medium quality (Table 1 and S2 File). 2 studies were of low quality.

**Table 1:** Characteristics of included studies and their quality assessment.

Reference	Location	Type of study	Study duration	Number of cases/ controls^a^	NOS^b^ score
Bern 2002 [25]	Peru	Cohort	February 1995-December 1998	143 cases	6
Chacin-Bonilla 2008 [35]	Venezuela	Cross-sectional	Rainy season	67 cases	7
Cruz 1988 [16]	Guatemala	Cohort	July 1985-June 1986	20 cases	5
Javier Enriquez 1997 [23]	Mexico	Cross-sectional	June 1988-May 1989	26 cases	4
Katsumata 1998 [17]	Indonesia	Case control (hospital study) & cross-sectional (community study)	August 1992-July 1993 for hospital study) & December 1992-March 1993 (rainy season) and (June-July 1993 (dry season) for community study	26 cases/ 1043 controls (hospital study) & 49 cases (community study)	7
Khan 2004 [18]	Bangladesh	Case control	May 2001-August 2002	46 cases/ 46 controls	3
Molbak 1994 [26]	Guinea-Bissau	Case control	June 1985-May 1988	125 cases/ 125 controls	6
Morse 2008 [19]	Malawi	Case control	January 2001-December 2002	24 cases/ 72 controls	5
Newman 1999 [36]	Brazil	Cohort	August 1989-August 1993	58 cases	7
Omoruyi 2011 [20]	South Africa	Case control	April 2009-January 2010	96 cases/ 20 controls	3
Pederson 2014 [34]	Tanzania	Cohort	6 months	102 cases	8
Pereira 2002 [37]	Brazil	Cross-sectional	August 1998-May 1999	83 cases	4
Salyer 2012 [24]	Uganda	Cross-sectional	May-June 2007	35 cases	5
Sarker 2014 [21]	India	Nested case control	2008–2013	406 cases/ 174 controls	7
Suarez Hernandez 1999 [22]	Cuba	Case control	Unknown	37 cases/ 185 controls	4

^a)^: # cases/ controls if only applicable for case control studies. For cross-sectional and cohort studies, only number of cases is included.

^b)^: Newcastle Ottawa Score (NOS) for Quality Assessment.

For each study, odds ratios (or equivalent) for each relevant risk factor were extracted and are detailed in S1 Table. The most frequently reported *Cryptosporidium* transmission pathways are: i) person to person (related to living in overcrowded accommodation, diarrhoea in the household or attending nursery), ii) water related (drinking poor quality water or contact with contaminated water bodies for hygiene or leisure purposes), iii) environmental transmission (animal contact, feces contaminated soil, farming or sewage proximity), iiii) inadequate sanitation (lack of toilet and/or open defecation) and iiiii) hygiene (hand, food or household). Details of transmission pathways are provided in S2 Table. Though not a transmission pathway, breastfeeding was investigated in 10/15 studies and as such was included in the analysis. When ≥ 4 studies reported on a risk factor, it was then included in the meta-analysis. This was the case for overcrowding, household diarrhoea, poor quality drinking water, animal contact, open defecation/ lack of toilet and breastfeeding (S2 Table).

### Animal contact

**Fig 2:**
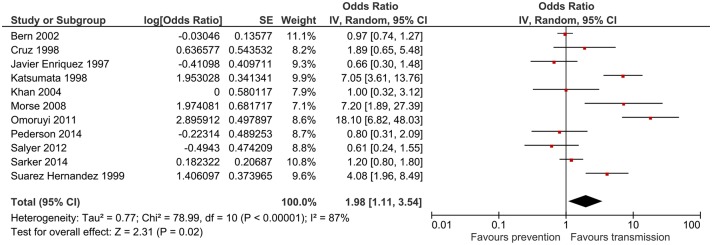
Meta-analysis for animal contact.

Animal contact was investigated in all 15 included studies. The type of animal species (when available) is provided in S1 Table. Information needed for meta-analysis could be extracted from 11/15 studies. While the majority of studies found that contact with animals is associated with increased risk of cryptosporidiosis [16–22], a few reported a protective effect. The combined odds ratio was 1.98 (95%CI: 1.11–3.54) p = 0.02 (Fig 2). Heterogeneity was substantial with I^2^ score of 87%.

### Poor drinking water quality

**Fig 3:**
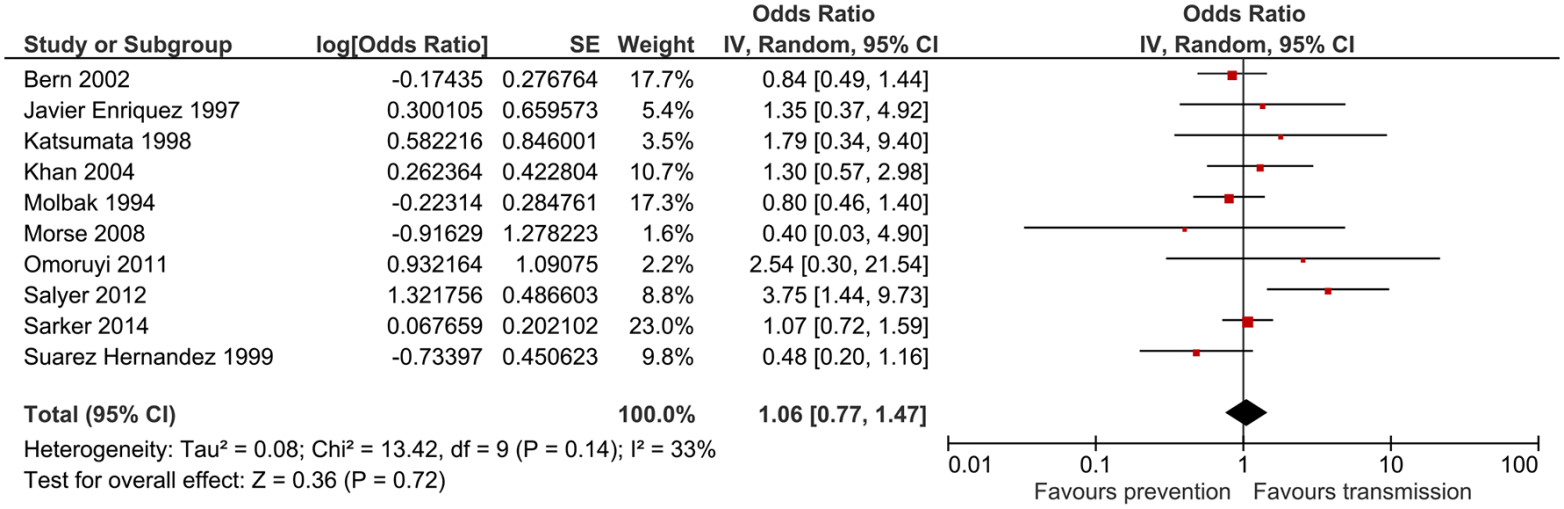
Meta-analysis for poor drinking water quality.

The impact of non-piped drinking water on cryptosporidiosis was investigated in all 15 studies. Meta-analysis was possible for 10 studies. The combined odds ratio was 1.06 (95%CI: 0.77–1.47), which was not significant (Fig 3). This was due to conflicting results between studies, with 6 studies reporting that non-piped water is a risk factor [17, 18, 20, 21, 23, 24], while 4 studies considered it to be protective [19, 22, 25, 26]. Heterogeneity was moderate (I^2^ = 33%).

### Open defecation/ lack of toilet facility

**Fig 4:**
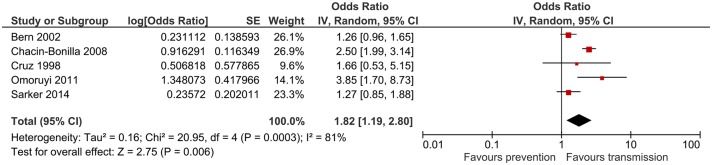
Meta-analysis for open defecation/ lack of toilet facility.

This risk factor was investigated in 7 studies. The combined odds ratio from 5 studies was 1.82 (95%CI: 1.19–2.8) p = 0.006 (Fig 4). Heterogeneity was substantial (I^2^ = 81%). Despite the relatively moderate combined risk associated with open defecation, all studies consistently showed increased cryptosporidiosis risk.

### Overcrowded living conditions

**Fig 5:**
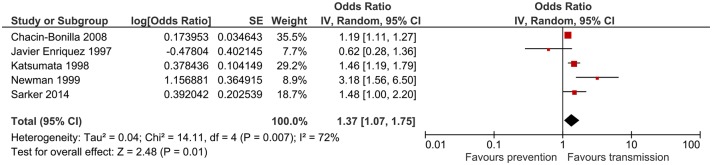
Meta-analysis for overcrowded living conditions.

Overcrowding was reported as a risk factor for *Cryptosporidium* infection in 7 studies. The combined odds ratio based on 5 studies was 1.37 (95%CI: 1.07–1.75) p = 0.01 (Fig 5). Heterogeneity was substantial (I^2^ = 72%). Only one study reported that overcrowding is protective [23].

### Diarrhoea in household

**Fig 6:**
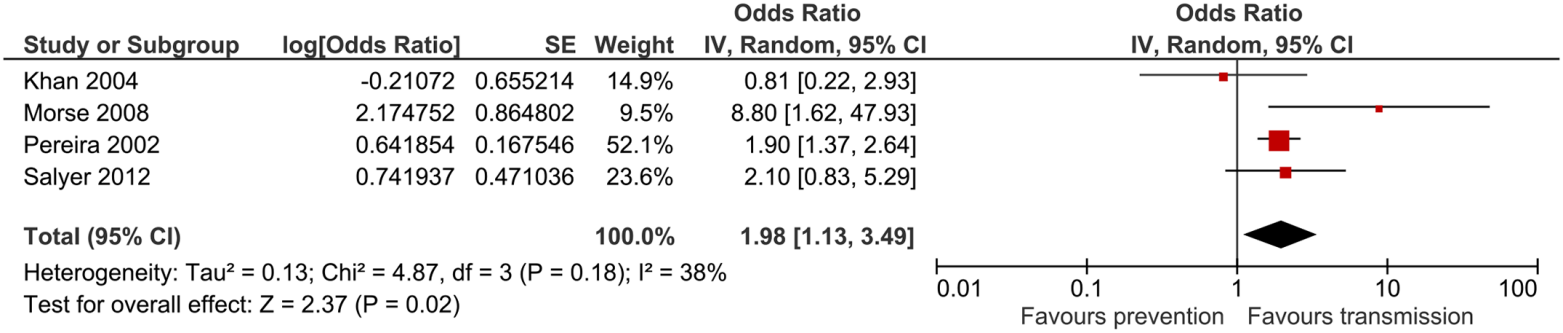
Meta-analysis for diarrhoea in household.

Household diarrhoea was a cryptosporidiosis risk factor in 4 studies, all of which were included in the meta-analysis. The combined odds ratio was 1.98 (95%CI: 1.13–3.49), which was statistically significant (Fig 6). Heterogeneity was moderate (I^2^ = 38%). One study reported that diarrhoea in the household was protective [18].

### Breastfeeding

**Fig 7:**
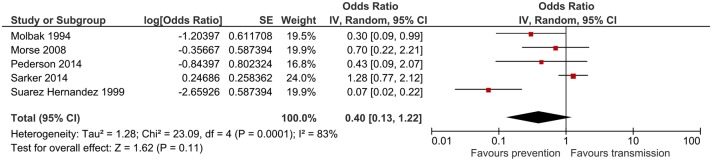
Meta-analysis for breastfeeding.

Breastfeeding was investigated in 10 studies. Meta-analysis was restricted to 5 studies that provided the required information. The combined odds ratio was 0.4 (95%CI: 0.13–1.22) suggesting a protective overall effect (Fig 7). However, this was not statistically significant. Heterogeneity was substantial (I^2^ = 83%). Only one study reported that breastfeeding was conducive to acquiring cryptosporidiosis in infants [21].

For each exposure, publication bias was assessed using funnel plots. All funnel plots had a symmetrical shape suggesting minimal publication bias (S1 Fig). Calculation of population attributable fraction (PAF) was possible for 4/5 risk factors. Breastfeeding was protective and therefore no PAF was calculated. Crowding was responsible for 18% of cases (95%CI 4–29%) based on 3 studies (Table 2). Open defecation was attributable to 17% of cases (95%CI 6–25%) (based on 4 studies), while animal contact accounted for 25% of cases (95%CI 5–36%) (8 studies). Poor drinking water quality was responsible for 2% of cases (95%CI—10%, 11%) (based on 8 studies).

**Table 2:** Risk factors identified, their pooled odds ratios and PAF.

Risk Factor	Pooled odds ratio	95% CI	P value	PAF
***Animal contact***	1.98	[1.11–3.54]	0.02	25% of cases (95%CI 5–36%)
***Poor drinking water quality***	1.06	[0.77–1.47]	0.72	2% of cases (95%CI—10%, 11%)
***Lack of toilet facility***	1.82	[1.19–2.8]	0.006	17% of cases (95%CI 6–25%)
***Overcrowded living conditions***	1.37	[1.07–1.75]	0.01	18% of cases (95%CI 4–29%)
***Diarrhoea in household***	1.98	[1.13–3.49]	0.02	NP
***Breastfeeding***	0.4	[0.13–1.22]	0.11	NA

PAF: Population attributable fraction. NP: not possible to calculate. NA: not applicable.

## Discussion

This systematic review aimed to identify the most frequently reported risk factors for *Cryptosporidium* infection from LMICs based on good quality studies. Although the search strategy retrieved > 3000 papers, only 15 studies were of acceptable quality warranting inclusion. The pitfall of this strategy is the exclusion of relevant risk factors and decreasing the power of meta-analysis by including fewer studies. Nevertheless, this review identified six risk factors that are likely to be the main drivers of *Cryptosporidium* infection in LMICs. Animal contact had the highest combined odds ratio 1.98 (95%CI: 1.11–3.54), which was statistically significant. This is in accordance with reviews from developed countries where animal contact/ farm visits/ petting zoo visits were significantly associated with acquiring cryptosporidiosis [7, 11]. Diarrhoea in the household was associated with a similar *Cryptosporidium* infection risk, pooled odds ratio 1.98 (95%CI: 1.13–3.49), which was also statistically significant. Case contact is understandably a risk factor for transmitting any infectious disease and this is relevant in both developed and developing nations. However, as the majority of *Cryptosporidium* infections are associated with mild self-limiting symptoms in healthy adults and could be asymptomatic in children, the number of diagnosed cases are substantially underestimated, contributing to further *Cryptosporidium* transmission unless proper hand hygiene and prevention measures are implemented. Similarly, overcrowded living conditions are associated with an increased risk of *Cryptosporidium* infection, pooled odds ratio 1.37 (95%CI: 1.07–1.75). Another person to person transmission pathway is children attending nursery. This risk factor was not assessed in many studies and therefore could not be included in the meta-analysis. This was also the case in developed countries, where only a few studies reported that nursery attendance and changing nappies are risk factors for *Cryptosporidium* infection [10].

Poor WASH (Water, Sanitation and Hygiene) conditions are paramount to the spread of *Cryptosporidium* and other gastrointestinal infections. The search strategy was not restricted to focus on WASH. Nevertheless, lack of appropriate sanitation/ open defecation was associated with a significant risk of acquiring cryptosporidiosis, pooled odds ratio 1.82 (95%CI: 1.19–2.8) based on 5 studies. This result is comparable to the systematic review by Speich and colleagues, who reported that lack of sanitation was associated with *Cryptosporidium* infection risk, pooled odds ratio 1.47 (95%CI: 0.37–5.88) based on 5 studies [27]. Interestingly, the 5 papers included in our systematic review and the one by Speich and colleagues were different, yet the associated risk was comparable. Our search strategy retrieved the papers included in Speich and colleagues, but these were not included as they considered less than 3 risk factors and/ or the data were missing from the full text. Indeed Speich and colleagues reported that they contacted some of the authors to obtain data that was collected but not analysed/ presented in the full text. This was an issue that we encountered while conducting this systematic review as several authors reported the investigation of several risk factors, which were omitted in the results section. Improving sanitation coverage is one of the aims of the Sustainable Development Goals. Though the number of people practising open defecation globally decreased from 38% to 25% between 1990 and 2015, there are currently 946 million people lacking sanitation worldwide (1 in 8) [28]. Open defecation is a clear indicator of extreme poverty and is associated with significant disease burden.

In this systematic review, poor drinking water was not associated with *Cryptosporidium* infection, however, this was not statistically significant. This was due to the contradicting findings of the included studies and wide confidence intervals. We considered the absence of piped water an indicator of poor drinking water quality. However, this is not necessarily true. The microbiological quality of spring and well water could be satisfactory for the majority of time unless contamination events occur. Indeed, many of the papers retrieved by our search strategy highlighted the increased risk of *Cryptosporidium* infection in the wet season and/or following extreme rain events. Regular consumption of contaminated drinking water, though not recommended from a public health view, could be associated with building protective immunity [29, 30]. Furthermore, drinking water could be a minor transmission pathway in endemic settings. Indeed, in a quasi-experimental study in India, drinking bottled water was not associated with reduced risk of cryptosporidiosis in children [31].

Breastfeeding (or lack of) was investigated in numerous studies that focused on childhood cryptosporidiosis. Breastfeeding was associated with a protective effect, however, this was not statistically significant. The protection potentially conferred by breastfeeding could be due to the passive immunity acquired through ingestion of *Cryptosporidium* specific antibodies in breast milk [32]. Additionally, bottle feeding was found to increase the risk of cryptosporidiosis [33], most likely due to one or a combination of the following factors: poor water quality, lack of sterilisation and substandard hand and household hygiene. Indeed, one study found that washing hands before infant feeding was associated with a significant cryptosporidiosis risk, multivariate adjusted odds ratio 5.02 (95%CI: 1.11–22.78) [34], which demonstrates the poor quality of water used for drinking and hand washing.

The main limitation of this systematic review is the small number of studies included. As strict inclusion criteria were applied, a large number of papers that could have added to the body of evidence were excluded. This was because they had a small number of *Cryptosporidium* cases, explored less than 3 risk factors (excluding age, gender, rural/ urban residence, malnutrition) or reported their results incompletely or inappropriately for inclusion in meta-analysis. This resulted in a small number of studies for each risk factor, which in turn reduced the power of meta-analyses performed. Additionally, this could have inevitably resulted in the exclusion of some other relevant risk factors for *Cryptosporidium* infection, that are investigated less frequently and/or not in conjunction with well-known transmission pathways. The main limitations of some of the included studies are the small number of cryptosporidiosis cases and poor quality in terms of study design and duration, number of exposures investigated and data reporting. Another shortcoming was that several papers presented risk factors for diarrhoeal diseases in general without categorisation/ sub group analysis for each etiologic agent. Some did not even seek to diagnose diarrhoeal pathogens. This limits the usefulness of such epidemiological studies and hinders the identification of relevant risk factors and the formulation of specific prevention measures. A heterogeneity between the included studies was noted. While, the majority of studies used diarrhoea free, *Cryptosporidium* negative control groups, some used diarrhoeal subjects that were *Cryptosporidium* negative. Both sets were undistinguishably included in the meta-analysis, however, combining them would introduce bias in the overall risk associated with each exposure.

In summary, this systematic review identified animal contact, diarrhoea in the household and open defecation as the most relevant risk factors associated with *Cryptosporidium* infection in LMICs. Improving sanitation coverage is one of the Sustainable Development Goals and progress is likely to happen despite the high number of people still practising open defecation globally. Animal contact and case contact/ household diarrhoea are relevant for both developed and developing countries and prevention measures should include awareness campaigns and better hand hygiene. Other relevant risk factors could have been omitted from the systematic review because of the paucity of data and poor quality of several studies. Considering the significant morbidity and mortality of cryptosporidiosis in sub-Saharan Africa and South Asia, especially for under 5 years (and HIV+), strategies to reduce the prevalence and burden of cryptosporidiosis and other gastrointestinal opportunistic diseases should be prioritised and offered adequate funding.

## Supporting information

S1 File PRISMA checklist. (DOC)

S2 File Newcastle-Ottawa quality assessment. (DOCX)

S1 Table *Cryptosporidium* risk factors identified in all included studies presented as odds ratios (unless stated otherwise), with 95% confidence intervals and p value. (DOCX)

S2 Table *Cryptosporidium* transmission pathways and most frequently reported risk factors in the included studies. (DOCX)

S1 Fig Funnel plots of studies included in the meta-analysis. (TIFF)
