# Supplementary material for: Risk factors for Cryptosporidium infection in low and middle income countries: A systematic review and meta-analysis
Source: PLoS Negl Trop Dis. 2018 Jun 7;12(6):e0006553. doi: 10.1371/journal.pntd.0006553 (PMC6014672; doi:10.1371/journal.pntd.0006553)
Supplement: S2 File — (DOCX) [file pntd.0006553.s002.docx]

**Newcastle-Ottawa Quality Assessment Scale**

**Case-control and cross-sectional studies**

**Selection**

1) Is the case definition adequate?

a) Yes, with independent validation: Laboratory confirmed *****

b) No: Self-reported or no description

2) Representativeness of the cases

a) Consecutive or obviously representative series of cases *****

b) Potential for selection biases or not stated

3) Selection of Controls

a) Community controls *****

b) Hospital controls or no description

4) Definition of Controls

a) No history of disease i.e. no diarrhea *****

b) No description of source

**Comparability**

1) Comparability of cases and controls

a) Yes: Adjusted analysis performed *****

b) No adjusted analysis

**Exposure**

1) Ascertainment of exposure

a) Structured interview where blind to case/control status *****

b) Interview not blinded to case/control status, self-report or no description

2) Same method of ascertainment for cases and controls

a) Yes *****

b) No

3) Non-Response rate

a) Same rate for both groups *****

b) Rate different and no designation

**Cohort studies**

**Selection**

1) Representativeness of the exposed cohort?

a) Truly or somewhat representative of the community *****

b) Selected group of users or no description

2) Selection of the non exposed cohort

a) Drawn from the same community as the exposed cohort *****

b) Drawn from a different source or no description

3) Ascertainment of exposure

a) Structured interview *****

b) Written self-report or no description

4) Demonstration that the outcome of interest was not present at the start of the study

a) Yes: if *Cryptosporidium* screening was performed at the start of the study or if following birth cohort *****

b) No

**Comparability**

1) Comparability of cohorts

a) Yes: Adjusted analysis performed *****

b) No adjusted analysis

**Outcome**

1) Assessment of outcome

a) Independent blind assessment: if *Cryptosporidium* infection confirmed by laboratory testing *****

b) Self-report or no description

2) Was follow up long enough for outcomes to occur?

a) Yes: if any *Cryptosporidium* cases were detected during the study *****

b) No

3) Adequacy of follow up of cohorts

a) Yes: subjects lost to follow up unlikely to introduce bias, if <20% subjects lost after start of the study *****

b) No: > 20% subjects lost after the start of the study or no description

**Quality assessment of the included studies**

| **CASE CONTROL AND CROSS SECTIONAL STUDIES** | | | | | | | | | |
| --- | --- | --- | --- | --- | --- | --- | --- | --- | --- |
|  | **Selection** | | | | **Comparability** | **Exposure** | | |  |
|  | Case Definition | Representativeness of Cases | Selection of Controls | Definition of Controls | Adjusted analysis | Ascertainment | Same method for cases and controls | Non response rate | **# STARS (out of 8)** |
| Chacin-Bonilla 2008 | * | * | * | * | * | * | * |  | 7 |
| Javier Enriquez 1997 | * | * |  |  |  | * | * |  | 4 |
| Katsumata 1998 | * | * | * | * | * | * | * |  | 7 |
| Khan 2004 | * | * |  |  |  |  | * |  | 3 |
| Molbak 1994 | * | * | * | * |  | * | * |  | 6 |
| Morse 2008 | * |  | * | * | * |  | * |  | 5 |
| Omoruyi 2011 | * |  |  |  |  | * | * |  | 3 |
| Pereira 2002 | * | * |  |  |  | * | * |  | 4 |
| Salyer 2012 | * |  | * |  | * | * | * |  | 5 |
| Sarker 2014 | * | * | * | * | * | * | * |  | 7 |
| Suarez Hernandez 1999 | * |  |  | * |  | * | * |  | 4 |
| **COHORTS STUDIES** | | | | | | | | | |
|  | **Selection** | | | | **Comparability** | **Outcome** |  |  |  |
|  | Representativeness of cohort | Selection of non exposed cohort | Ascertainment | Outcome not present at start of study | Adjusted | Assessment | Follow up duration | Adequacy of follow up | **# STARS (out of 8)** |
| Bern 2002 | * | * | * |  | * | * | * |  | 6 |
| Cruz 1988 | * | * | * |  |  | * | * |  | 5 |
| Newman 1999 |  | * | * | * | * | * | * | * | 7 |
| Pederson 2014 | * | * | * | * | * | * | * | * | 8 |
