# Supplementary material for: Risk factors for Cryptosporidium infection in low and middle income countries: A systematic review and meta-analysis
Source: PLoS Negl Trop Dis. 2018 Jun 7;12(6):e0006553. doi: 10.1371/journal.pntd.0006553 (PMC6014672; doi:10.1371/journal.pntd.0006553)
Supplement: S1 Table — (DOCX) [file pntd.0006553.s003.docx]

S1 Table: *Cryptosporidium* risk factors identified in all included studies presented as Odds ratios (unless stated otherwise), with 95% confidence intervals and p value. RR: Risk Ratio, HR: Hazard ratio

| **Reference** | **Risk Factors** | **Odds Ratio** | **95% CI** | **P** |
| --- | --- | --- | --- | --- |
| Bern 2002 | Defecates in field | RR= 1.26 | 0.97-1.67 | 0.08 |
| Bern 2002 | Water source outside house | RR=0.84 | 0.49- 1.45 | 0.54 |
| Bern 2002 | Contact with animals | RR= 0.97 | 0.74- 1.26 | 0.82 |
| Chacin-Bonilla 2008 | Crowding (> 2 people per bedroom) | 1.19 | 1.1- 1.26 | < 0.05 |
| Chacin-Bonilla 2008 | No sanitary facility/ Open defecation | 2.5 | 1.99- 3.14 | < 0.01 |
| Chacin-Bonilla 2008 | Contact with feces contaminated soil | 2.68 | 2.02- 3.56 | < 0.01 |
| Cruz 1988 | Water source Public/piped | RR=1.00 |  |  |
| Cruz 1988 | Excreta disposal Pit/toilet | RR=1.66 | 0.60-5.78 |  |
| Cruz 1988 | Domestic animals in the house | RR=1.89 | 0.69-5.81 |  |
| Cruz 1988 | Consumption of solids and/or gruels | RR=2.07 | 0.58-9.50 |  |
| Cruz 1988 | Breast-feeding | RR=6.74 |  |  |
| Javier Enriquez 1997 | Water source (well versus tap) | 1.35 | 0.37-4.91 | 0.64 |
| Javier Enriquez 1997 | Breastfeeding | 0.17 | 0.02-1.29 | 0.0537 |
| Javier Enriquez 1997 | 1 Room Home | 0.62 | 0.277-1.34 | 0.245 |
| Javier Enriquez 1997 | Domestic animals | 0.66 | 0.29-1.48 | 0.313 |
| Katsumata 1998 | Contacts with cats | 7.05 | 3.61–13.76 |  |
| Katsumata 1998 | Drinking untreated water | 1.79 | 0.34–9.37 |  |
| Katsumata 1998 | Bathing in public bath | 1.72 | 0.77–3.85 |  |
| Katsumata 1998 | Crowding | 1.46 | 1.19–1.79 |  |
| Khan 2004 | Contact with animals | 1 | 0.32-3.11 | 1 |
| Khan 2004 | Well water consumption vs mains | 1.3 | 0.57-2.99 | 0.52 |
| Khan 2004 | Other family members with diarrhea | 0.81 | 0.22-2.87 | 0.74 |
| Khan 2004 | Food preparation within 10 yards of animal | 0.61 | 0.23-1.63 | 0.33 |
| Molbak 1994 | Breastfeeding | 0.3 |  | 0.02 |
| Molbak 1994 | Storage of cooked food | 2.2 |  | 0.005 |
| Molbak 1994 | Presence of soap | 1.7 |  | 0.08 |
| Molbak 1994 | Water from well | 0.8 |  | 0.48 |
| Molbak 1994 | Child open defecation | 1 |  | 0.88 |
| Molbak 1994 | Pigs in own household | 2.3 |  | 0.002 |
| Molbak 1994 | Breast feeding (Multiregression) | 0.3 | 0.1- 1.1 | 0.048 |
| Molbak 1994 | Consumption of stored food (multiregression) | 1.8 | 1.0-3.3 | 0.018 |
| Molbak 1994 | Pigs in household (multiregression) | 2.5 | 1.4-4.7 | 0.004 |
| Molbak 1994 | Dogs in household (multiregression) | 2.1 | 1.0-4.2 | 0.051 |
| Morse 2008 | Diarrhoea in other household members | 8.8 | 1.8 – 53.4 | 0.008 |
| Morse 2008 | Bathing in a river | 6.7 | 1.1 – 23.8 | 0.04 |
| Morse 2008 | Ownership of pigs | 7.2 | 1.9 – 27.5 | 0.004 |
| Morse 2008 | Drinking stored water | 0.4 | 0.01 – 1.5 | 0.17 |
| Morse 2008 | Breastfeeding | 0.7 | 0.2 – 2.0 | 0.5 |
| Newman 1999 | Crowded residence | HR =3.18 | 1.55-6.48 | 0.001 |
| Omoruyi 2011 | Drinking river water vs tap water | 2.54 | 0.3-21.58 | 0.39 |
| Omoruyi 2011 | Contact with farm animals | 18.1 | 6.82-48.02 | 0.0001 |
| Omoruyi 2011 | Using pit vs flush toilet | 3.85 | 1.7-8.75 | 0.001 |
| Pederson 2014 | Maternal Cryptosporidium infection at month 1 | 3.18 | 1.01- 9.99 | 0.047 |
| Pederson 2014 | Exclusive breastfeeding at month 3 | 0.43 | 0.09-2.09 | 0.29 |
| Pederson 2014 | Owns animals | 0.8 | 0.31-2.11 | 0.65 |
| Pederson 2014 | Washes hands (prior to infant feeding) | 1.72 | 0.68-4.33 | 0.24 |
| Pederson 2014 | Maternal Crypto infection at month 1 (multivariate adjusted) | 3.4 | 0.88-13.06 | 0.075 |
| Pederson 2014 | Partial breastfeeding at month 3 (multivariate adjusted) | 0.32 | 0.05–2.08 | 0.23 |
| Pederson 2014 | Owns animals (multivariate adjusted) | 0.75 | 0.19-2.94 | 0.67 |
| Pederson 2014 | Washes hands prior to infant feeding (multivariate adjusted) | 5.02 | 1.11-22.78 | 0.036 |
| Pereira 2002 | Day care attendance | 2.1 | 1.1–3.8 | 0.06 |
| Pereira 2002 | Children with diarrhea in household | 1.9 | 1.4–2.7 | 0.001 |
| Pereira 2002 | Recreational contact with bodies of water | 3.4 | 1.1–10.6 | 0.1 |
| Pereira 2002 | Household 50m of raw surface water or sewage effluent | 0.45 | 0.26–0.78 | 0.01 |
| Salyer 2012 | Worked in agricultural fields | 0.87 | 0.34- 2.26 | 0.812 |
| Salyer 2012 | Fetched water from open water source | 3.75 | 1.44- 9.7 | 0.008 |
| Salyer 2012 | Tended livestock | 0.61 | 0.24- 1.54 | 0.4195 |
| Salyer 2012 | Another positive person in household | 2.1 | 0.83- 5.26 | 0.13 |
| Salyer 2012 | Positive livestock in household | 6.75 | 0.67- 67.4 | 0.099 |
| Salyer 2012 | Fetching water from an open source (multiregression adjusted) | 2.96 | 1.1–7.99 | 0.032 |
| Sarker 2014 | Exclusive breastfeeding ≥ 6 months | 1.28 | 0.77-2.12 | 0.336 |
| Sarker 2014 | Presence of older sibling | 1.54 | 1.07-2.21 | 0.018 |
| Sarker 2014 | Overcrowding | 1.48 | 0.99-2.19 | 0.05 |
| Sarker 2014 | Good household hygiene | 0.86 | 0.61-1.23 | 0.423 |
| Sarker 2014 | Never boiling drinking water | 1.07 | 0.72-1.59 | 0.71 |
| Sarker 2014 | Presence of toilet | 0.79 | 0.53-1.17 | 0.242 |
| Sarker 2014 | Toilet used by all family members | 0.75 | 0.51-1.09 | 0.133 |
| Sarker 2014 | Direct contact with animals | 1.2 | 0.8-1.8 | 0.377 |
| Sarker 2014 | Presence of cows in the house/handling of cow dung | 1.3 | 0.86-1.95 | 0.212 |
| Sarker 2014 | Open sewage channel within 10 m of house | 0.98 | 0.66-1.45 | 0.92 |
| Sarker 2014 | Open air defecation area within 50m of the house | 0.92 | 0.51-1.66 | 0.784 |
| Sarker 2014 | Older siblings in the house (multivariate logistic regression) | 1.88 | 1.26–2.80 | 0.002 |
| Sarker 2014 | Boiling drinking water (multivariate logistic regression) | 0.49 | 0.25–0.97 | 0.041 |
| Sarker 2014 | Toilet used by all family members | 0.63 | 0.41–0.97 | 0.036 |
| Suarez Hernandez 1999 | Breastfeeding | 0.07 | 0.02-0.20 | < 0.0001 |
| Suarez Hernandez 1999 | Contacts with animals | 4.08 | 1.96-8.49 | 0.0002 |
| Suarez Hernandez 1999 | Not boiling drinking water | 0.48 | 0.2-1.17 | 0.1 |
| Suarez Hernandez 1999 | Washing hands before preparing child’s food | 0.39 | 0.19-0.81 | 0.01 |
| Suarez Hernandez 1999 | Attending nursery | 1.72 | 0.67-4.4 | 0.25 |
