# Supplementary material for: Risk factors for Cryptosporidium infection in low and middle income countries: A systematic review and meta-analysis
Source: PLoS Negl Trop Dis. 2018 Jun 7;12(6):e0006553. doi: 10.1371/journal.pntd.0006553 (PMC6014672; doi:10.1371/journal.pntd.0006553)
Supplement: S2 Table — (DOCX) [file pntd.0006553.s004.docx]

S2 Table: *Cryptosporidium* transmission pathways and most frequently reported risk factors in the included studies. Breastfeeding was investigated in 10/15 studies and as such was included in the analysis. The number of studies asking about each individual risk factor and the number (and %) of statistically significant risk factors are presented. When > 4 studies reported on a risk factor, it was then included in the meta-analysis.

Y in box means study found the risk factor significant. Shaded green boxes indicate the study asked about the risk factor.
