# Supplementary figures and images for: Risk factors for Cryptosporidium infection in low and middle income countries: A systematic review and meta-analysis
Source: PLoS Negl Trop Dis. 2018 Jun 7;12(6):e0006553. doi: 10.1371/journal.pntd.0006553 (PMC6014672; doi:10.1371/journal.pntd.0006553)

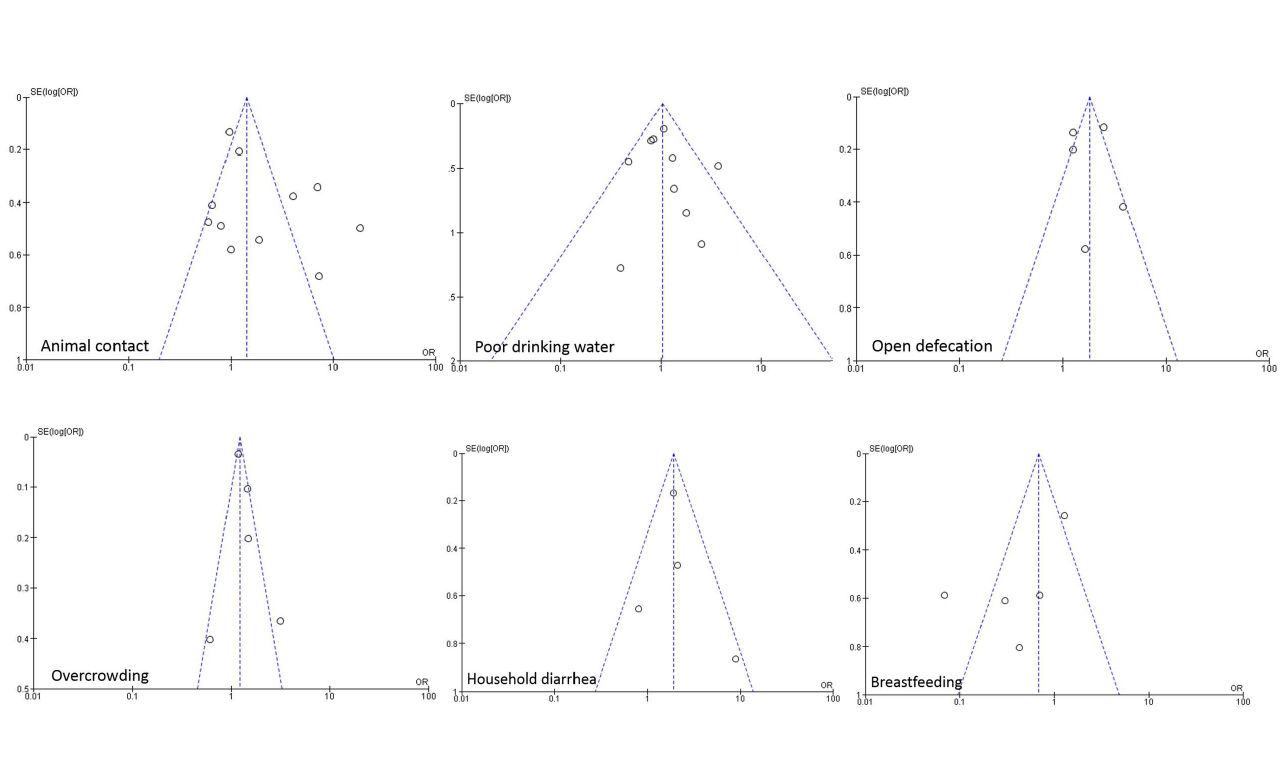

Supplement: S1 Fig — (TIFF) [file pntd.0006553.s005.tiff]
